# Hematological Markers to Predict Enterectomy Versus Enterotomy in Dogs with Foreign-Body Intestinal Obstruction: Preliminary Data

**DOI:** 10.3390/ani15010024

**Published:** 2024-12-25

**Authors:** Lorena Espadas González, Eva M. Pérez-Merino, Irene Cantalejo Rodrigo, Jesús M. Usón-Casaús, Nieves Pastor Sirvent

**Affiliations:** 1 Department of Animal Medicine, Facultad de Veterinaria, Universidad de Extremadura, Avenida de la Universidad s/n, 10003 Cáceres, Spain; lorenaeg@unex.es (L.E.G.); jmuson@unex.es (J.M.U.-C.); nievespastor@unex.es (N.P.S.); 2 Veterinary Teaching Hospital, Universidad de Extremadura, Avenida de la Universidad s/n, 10003 Cáceres, Spain; irenecr27@gmail.com

**Keywords:** dog, blood-derived ratios, intestinal resection, foreign-body obstruction

## Abstract

In dogs with intestinal obstruction due to foreign bodies (FBOs), the potential of preoperative hematological parameters in predicting enterectomy (EE) versus enterotomy (EO) has not been previously studied. In this retrospective analysis, 102 dogs with FBO were categorized into EO (n = 60) and EE (n = 42) groups based on the procedure performed. The presurgical white blood cells (WBCs), neutrophils, lymphocytes, platelet counts, neutrophil-to-lymphocyte ratio (NLR), platelet-to-lymphocyte ratio (PLR), and systemic immune-inflammation index (SII) were compared. The area under the curve (AUC) and cutoffs were determined. WBCs (*p* = 0.001), lymphocytes (*p* = 0.006), neutrophils, NLR, SII (*p* = 0.0001), and PLR (*p* = 0.043) were significantly associated with EE, but platelets were not (*p* = 0.226). NLR had the greatest AUC (0.828 [0.732–0.924]), while lymphocytes and PLR showed a low predictive validity. The identified cutoff values included 19.85 × 10^3^/µL for WBCs, 14.19 × 10^3^/µL for neutrophils, 12.7 for NLR, and 2812.59 × 10^3^ for SII. Values exceeding these thresholds increased the EE risk by 6.40-, 28.00-, 15.96-, 3.25-, and 12.42-fold, respectively. Elevated preoperative WBCs, neutrophils, SII, and especially NLR could help to predict the need for intestinal resection, aiding surgical planning and improving outcomes.

## Simple Summary

This study examines the use of preoperative blood markers to predict the type of surgery needed in dogs with gastrointestinal blockages due to foreign objects. In this situation, veterinarians must choose intraoperatively between two procedures: enterotomy (EO), a simpler surgery, or enterectomy (EE), a more complex surgery required if tissue damage is severe. EE carries higher risks of complications, mortality, and expenses. Therefore, early, reliable indicators that guide veterinarians to predict intestinal resection and help to set realistic expectations for pet owners are desirable. This study focused on complete blood-derived markers, known predictors of intestinal damage in humans. We tested these markers in 102 dogs, grouped by the surgery type they received (EO or EE). The results showed that white blood cell count, neutrophil count, and systemic immune-inflammation index levels were useful in predicting the need for EE, with the neutrophil-to-lymphocyte ratio providing the most accurate predictions. These blood tests are affordable, fast, and effective, giving veterinarians important information to improve patient care and communicate risks to pet owners more effectively.

## 1. Introduction

Intestinal obstruction caused by a foreign body accounts for 80% of all canine intestinal blockages [1,2]. Surgical options to address this issue include enterotomy (EO) or enterectomy (EE) with anastomosis, depending on the degree of bowel ischemia. These procedures differ in their complexity, overall success rates, and financial cost. EE is more challenging than EO and involves longer surgical and anesthesia times, extended hospital stays, increased costs, and a higher risk of complications [3]. Studies have shown that resection and anastomosis are associated with a greater risk of short bowel syndrome, leakage and dehiscence [4,5] and a higher likelihood of mortality compared to EO [6] when removing foreign bodies. The decision to perform one procedure over the other is made intraoperatively based on the assessment of intestinal viability. However, given the implications of each technique, any preoperative data that could aid in predicting intestinal resection would be valuable for guiding the owner’s expectations and enhancing patient management. One study attempted to correlate history, physical examination, radiographic and ultrasound findings, and presurgical blood biochemistry analysis with the likelihood of performing EO or EE in dogs. However, it did not examine the relationship between presurgical hematology reports and the selected surgical procedure [3].

Damage to the intestinal wall triggers a localized inflammatory response that can progress to a systemic inflammatory response, characterized by a decrease in lymphocyte count and an increase in neutrophils and platelets [7,8]. Therefore, hematological bioindicators such as the neutrophil-to-lymphocyte ratio (NLR), platelet-to-lymphocyte ratio (PLR), and systemic immune-inflammation index (SII) have been widely studied in humans for their effectiveness in detecting bowel ischemia or predicting small-bowel resection in cases of non-cancerous obstruction, including incarcerated hernia [9,10,11,12,13,14], intussusception [15], and adhesions [8,16].

However, to the authors’ knowledge, the association between these markers and the choice of surgical procedure for foreign-body-related intestinal obstruction in dogs has not yet been investigated. This study aims to evaluate the utility of preoperative blood analytes and the NLR, PLR, and SII indices in predicting the need for EO or EE in dogs with intestinal foreign-body obstructions.

## 2. Materials and Methods

### 2.1. Groups and Data Collection

This study was conducted as a retrospective case–control study. The electronic medical records from the Veterinary Teaching Hospital at the University of Extremadura (VTH-UEx) were reviewed for all dogs that underwent EO or EE due to mechanical obstruction from foreign-body ingestion between May 2019 and September 2024. Written consent to investigate and use these data was obtained from all owners of the dogs included in this study.

Upon admission, all dogs received a physical examination, hematology and biochemistry blood tests, abdominal radiographs, and ultrasonography. Once an intestinal foreign body was diagnosed and obstruction confirmed, emergency surgery was recommended.

Patients were classified into two groups—EO or EE—based on the procedure documented in the surgical report and performed. The exclusion criteria included incomplete surgical or hematology reports, obvious intestinal perforation and peritonitis at the time of surgery, immune-compromised status, other causes of small-bowel obstruction, or concurrent diseases. Dogs that underwent therapeutic endoscopy in conjunction with EO or EE were included. Experienced surgeons performed all procedures.

The recorded data included patient demographics, previous treatments before admission at the VTH, disease duration defined as the interval from the onset of clinical signs according to the owner and the admission and blood sampling at the VTH, time from diagnosis and surgery at the VTH, and the ASA score. The blood test results used for this study were those obtained at the VTH-UEx during the diagnostic phase.

A hematological analysis, including a white blood cell (WBC) differential, was conducted at admission as part of the diagnosis process using an IDEXX ProCyte Dx Analyzer (IDEXX B.V., Hoofddorp, The Netherlands). Absolute counts for platelets, neutrophils, and lymphocytes were recorded. Preoperative NLR and PLR were calculated from the differential count by dividing the absolute neutrophil and platelet counts by the absolute lymphocyte count, respectively. SII values were calculated using the platelet × neutrophil/lymphocyte count formula.

Since the reference values for these ratios vary between studies [17,18,19,20,21,22], a control group of healthy dogs admitted for routine sterilization at the Veterinary Teaching Hospital from January to May 2023 was used to establish reference values. These dogs were confirmed to be healthy based on their clinical history, physical examination, and hematobiochemical profile and they were not receiving any medications. The owners signed informed consent to use these data.

### 2.2. Statistical Analysis

Statistical analysis was conducted using the Statistical Package for Social Sciences (SPSS), version 27.0 (SPSS, Chicago, IL, USA). The Kolmogorov–Smirnov test was applied to assess normality. As the data were not normally distributed, descriptive statistics were reported as the median and range (minimum–maximum; standard deviation). Mann–Whitney U tests were used to determine significant differences between groups. A receiver operating characteristic (ROC) curve analysis was conducted to evaluate the ability of analytes and ratios to distinguish between EE and EO. The area under the curve (AUC) was calculated, and the optimal cutoff point for each parameter was identified using the Youden index (maximizing sensitivity and specificity). Odds ratios were calculated using these cutoff values. A *p*-value of less than 0.05 was considered statistically significant.

## 3. Results

**Figure 1:**
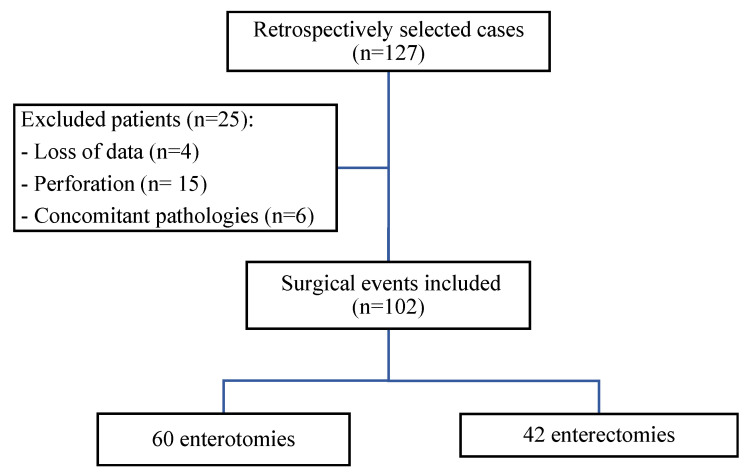
Flowchart determining case eligibility.

A total of 127 dogs that underwent EO or EE were included in this study. Twenty-five were excluded due to data loss, perforation, or other pathologies. Of the remaining 102 dogs that met the inclusion criteria, 60 underwent EO and 42 underwent EE (Figure 1).

The control group consisted of 40 dogs (20 male and 20 female) of various breeds, including 12 mixed breeds; 10 Spanish Greyhounds; four each of German Shepherds, Weimaraners, and Collies; and two each of medium Poodles, Pomeranians, and Spanish Podencos. The median age was 24 months (7–60; 13.98), and the median weight was 18.12 kg (3.8–37; 9.24). The group of dogs undergoing EO included 21 mixed breeds: four Labrador Retrievers; three each of Yorkshire Terriers and Beagles; two each of American Staffordshire Terriers, Weimaraners, Golden Retrievers, Spanish Mastiffs, German Shepherds, Pit Bull Terriers, Belgian Shepherds, and Bavarian Hounds; and one each of Bull Terriers, English Bulldogs, Poodles, Border Collies, Maltese Bichons, Italian Greyhounds, Cocker Spaniels, Pointers, Pomeranians, Shar Peis, Teckels, Spanish Podencos, and West Highland White Terriers. In total, 38 were male, and 22 were female, with a median age and weight of 61.00 (4–132; 38.56) months and 19.30 (1.6–62; 12.19) kilograms. The median ASA score was three (3–5; 0.72), and the median disease duration was four (1–15; 5.9) days. The EE group comprised 22 males and 20 females from different breeds: 15 mixed breeds; three each of German Shepherds and Beagles; two each of Border Collies, Labrador Retrievers, Pit Bull Terriers, West Highland White Terriers, and Spanish Podencos; and one each of Alaskan Malamutes, Maltese Bichons, Weimaraners, Spaniel Bretons, Pugs, Siberian Huskies, Spanish Mastiffs, Belgian Shepherds, Rottweilers, Irish Setters, and Teckels. The median age was 71.00 (6–168; 40.93) months, and the median weight was 17.90 (3.3–50; 11.55). The ASA score in this group was 4 (3–5; 0.72), and the median disease duration was 7 (1–20; 5.15) days.

The dogs in the control group were significantly younger than the dogs undergoing intestinal surgery (*p* = 0.001), but they did not differ in weight (*p* = 0.953). No significant differences were observed between the EO and EE groups in breed and sex distribution, age, or weight.

We could determine the administration of antiemetic, antidiarrheal, probiotics, and opioids before admission and blood sampling in 22 dogs (21.5%). Fasting or diets (chicken, rice, or digestive commercial diets) were recommended in 37 dogs (36.2%). Five dogs received antimicrobials, but the treatment finished more than a week before their admission at the VTH, and the symptoms relapsed when the antimicrobials were suppressed. Therefore, we decided to include them.

The median ASA score was significantly higher (*p* = 0.012), and the disease duration was longer (*p* = 0.007) in the EE group than in the EO group. The interval between blood sampling and diagnosis at the VTH and the surgery was always less than 24 h.

The investigated surgical reports indicated that the decision to perform EE was based on intraoperative findings, including dark intestinal coloration, the absence of arterial pulsation, lack of bleeding, and the absence of peristalsis.

Dogs undergoing intestinal surgery showed significantly higher WBC counts, neutrophil counts, NLR, PLR, SII, and lower lymphocyte counts (*p* = 0.0001) than healthy dogs. The EO group showed significantly higher platelet counts (*p* = 0.015) than the control group, but platelet numbers did not differ between the EE and the control group (*p* = 0.22).

After comparing both surgical groups, the WBC counts, neutrophil counts, NLR, PLR, and SII values were significantly higher, and the lymphocyte counts were lower in the EE group than in the EO group. No differences in platelet counts were found between the two surgical groups (Table 1).

**Figure 2:**
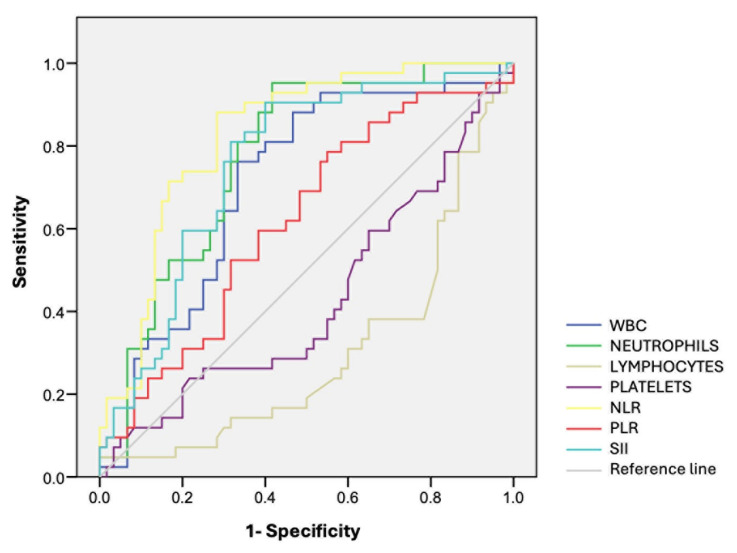
The receiver operating characteristics curve analysis for hematological biomarkers.

The area under the curve was statistically significant for all markers except for the platelet count. However, the ROC analysis revealed that the presurgical lymphocyte number was unable to distinguish between EO and EE. PLR showed the lowest AUC and was deemed a poor predictor (Figure 2).

WBCs, neutrophils, and SII were considered fair predictors of the need for EE, although the neutrophil cutoff showed low specificity. NLR emerged as the strongest predictor of intestinal resection, with the highest AUC (Table 2).

Risk analysis confirmed that values above the cutoff for WBCs (OR = 6.40: 95% CI: 2.62–15.58; *p* = 0.0001), neutrophils (OR = 28.00; 95% CI: 6.18–126.75; *p* = 0.0001), lymphocytes (OR = 0.17; 95% CI: 0.07–0.408; *p* = 0.0001), NLR (OR = 15.96; 95% CI: 5.42–47.05; *p* = 0.0001), PLR (OR = 3.25; 95% CI: 1.29–8.18; *p* = 0.01), and SII (OR = 12.42; 95% CI: 3.93–39.22; *p* = 0.0001) were significantly associated with an increased likelihood of enterectomy in dogs with intestinal obstructions caused by foreign bodies.

## 4. Discussion

The presence of a foreign body in the intestinal lumen can lead to progressive damage to the intestinal wall, initiating an inflammatory response that may transition from localized to systemic. Disruption of the mucosal barrier facilitates bacterial translocation, exposing the immune system to a spectrum of exogenous antigens. Neutrophils and macrophages phagocytize translocate bacteria, while antigens associated with tissue damage and pathogens activate toll-like receptors on mononuclear cells, triggering the release of acute-phase inflammatory proteins. These proteins coordinate the local inflammatory response, which can escalate to a systemic level. As the inflammatory cascade amplifies, multiple organs contribute to the production of inflammatory mediators, leading to widespread microvascular dysfunction beyond the initial site of obstruction. The severity, scope, and duration of this dysfunction can result in multi-organ failure. Systemic dissemination of locally generated inflammatory mediators via the circulatory and lymphatic systems underlies the development of systemic inflammatory response syndrome, while translocated bacteria may further exacerbate the condition by inducing sepsis [7,15]. The immune response to systemic inflammation typically involves neutrophilia, driven by neutrophil demargination and delayed apoptosis, and lymphopenia, resulting from lymphocyte redistribution and increased apoptosis [20]. Platelet counts may increase as part of the inflammatory response; however, thrombocytopenia is observed in critically ill or septic patients [8].

This retrospective study assessing the predictive value of blood analytes and hematological markers for intestinal resection and anastomosis in dogs with foreign-body-induced intestinal obstruction found that WBC, neutrophil count, and SII could serve as reliable indicators of the need for intestinal resection. Notably, the NLR demonstrated high sensitivity and specificity in identifying dogs likely to require enterectomy. These markers are cost-effective and can be measured rapidly, enabling clinicians to provide owners with better information regarding prognosis, estimated surgical costs, and the urgency of treatment.

While numerous studies in human medicine aim to predict intestinal ischemia, such research is limited in the veterinary field. Veterinary studies primarily focus on the complications or mortality associated with different intestinal surgeries [23,24]. In cases of canine foreign-body obstruction, one study investigating presurgical variables to differentiate EO from EE found that only an elevated heart rate and the severity of vomiting at presentation were associated with EE. However, that study conducted in dogs did not examine hematological markers as predictive indicators of intestinal resection [3], despite their proven utility in assessing surgery indication and the progression of non-malignant intestinal obstructions in humans [25]. In veterinary medicine, blood-derived markers have primarily been utilized for diagnostic and prognostic purposes in gastrointestinal conditions such as chronic enteropathy [19,20,26,27,28], acute diarrhea [29], and parvovirus infection [30] in dogs. This study offers new insights into the value of these markers for detecting ischemic intestinal injury in dogs, and, to the authors’ knowledge, no other similar studies address this issue.

In the present study, all blood cell counts in dogs with foreign-body intestinal obstruction, except for platelets, differed from healthy dogs. Elevated WBCs, neutrophils, NLR, PLR, and SII were associated with an increased risk of enterectomy, with the resection risk rising by 6.40-, 28.00-, 15.96-, 3.25-, and 12.42-fold, respectively, when these values exceeded the specified thresholds. Nevertheless, despite the differences observed between the surgical groups, lymphocytes and PLR were found to be ineffective predictors of enterectomy following ROC analysis. Moderate diagnostic accuracy was achieved for WBCs, neutrophils, and SII, while NLR demonstrated good accuracy.

Human studies yield inconsistent results regarding the predictive effects of blood cell counts and blood-derived ratios for small-bowel ischemia or resection. For instance, while one study reported significant differences in NLR and WBCs but not in neutrophil counts between patients who did or did not undergo bowel resection due to incarcerated groin hernia [14]; others have demonstrated that higher neutrophil counts are associated with an increased likelihood of bowel resection in the same context [13,31,32]. Intestinal resection has been linked to an elevated neutrophil number, NLR, PLR, and SII, though not to WBCs in cases of abdominal wall hernia [11]. However, another study found an association between resection and NLR, WBCs, and neutrophils [9] in the same clinical scenario. Resection was associated with a higher neutrophil count, NLR, and PLR but not with the lymphocyte count in children with intussusception [15] and, conversely, with low lymphocyte counts, along with NLR in cases of benign intestinal obstruction [25]. Platelet counts did not differ between the groups with resection and non-resection in any of these studies [9,11,14,15,25].

In cases of adhesional small-bowel obstruction, one study found that patients with ischemia or infarction had elevated preoperative NLR, PLR, WBCs, and neutrophils and lower lymphocyte counts than those without ischemia [8]. However, another study concluded that only NLR was predictive [16]. The ROC analyses in these studies did not demonstrate predictive value for PLR [8,16].

The variability in study findings regarding blood cell counts and their indicative value for resection or ischemia may arise from differences in population sizes, clinical scenarios, and study designs. One study emphasized the importance of blood sampling timing, noting that the initial neutrophil and lymphocyte counts taken at admission showed no association with ischemia in patients with adhesional small-bowel obstruction. However, these parameters became predictive when taken at the closest time prior to surgery. Conversely, platelet count at admission—but not preoperatively—was associated with ischemia. These findings suggest that as untreated ischemia progresses to necrosis, changes in blood cell counts become more pronounced, potentially affecting the predictive accuracy of these markers. For patients who underwent surgery within 24 h of admission, markers at admission were considered presurgical [8]. In our study, all surgeries were performed within 24 h of blood sampling. The disease duration varied between the EO and EE groups, as noted in a previous study [3]. This discrepancy likely contributes to the differences in hematological markers observed between the surgical groups, as certain gastrointestinal foreign bodies require more time to inflict the level of trauma and intestinal swelling necessary to devitalize bowel tissue [8]. In our study, we observed a longer disease duration (4 days [1,2,3,4,5,6,7,8,9,10,11,12,13,14,15] for EO and 7 days [1,2,3,4,5,6,7,8,9,10,11,12,13,14,15,16,17,18,19,20] for EE) than reported in another study (2 days [1,2,3,4,5,6,7,8] for EO and 4 days [1,2,3,4,5,6,7,8,9,10,11,12,13,14,15,16,17] for EE) [3], likely because we acted as a referral hospital in several cases. This difference may have affected our results and could help to explain the higher percentage of dogs undergoing EE in our study (41.1%) compared to previous reports (36.6% [3] and 19.4% [4]). All these findings suggest that the influence of timing on hematological marker assessments warrants further investigations.

Despite variations in blood cell results across studies, there is a consensus on the enhanced predictive ability of hematological ratios over their components. This was evident in the present study, where NLR outperformed both neutrophils and lymphocytes, while PLR and SII surpassed platelets and lymphocytes in ROC analysis, demonstrating a superior predictive capacity.

As can be seen, NLR is the most widely used hematological marker for predicting small-bowel ischemia or resection in human studies, and its effectiveness has been consistently validated. In contrast, PLR is used much less frequently, which may contribute to the inconsistent findings reported in the literature, while SII has rarely been employed. This study illustrates the utility of NLR and SII in predicting bowel resection in dogs, with NLR exhibiting a superior predictive capacity compared to other ratios, aligning with findings in similar human studies. However, we observed a poor predictive utility for PLR.

Different thresholds have been reported for these markers to differentiate between ischemia/resection and non-ischemia in humans. Two studies on intestinal resection due to incarcerated groin hernia identified NLR cutoffs of >11.5 [14] and >6.5 [13]. Although the first value closely aligns with our findings, neither study reported specificity or sensitivity. For bowel ischemia resulting from obstructive intestinal adhesions, NLR cutoffs of >7.4 (sensitivity, 85.2%; specificity, 60.3%) [8] and >6.8 (sensitivity, 78%; specificity, 65%) [16] have been documented. Bowel resection was associated with NLR cutoffs of >6.61 (sensitivity, 59.6%; specificity, 72.8%) [25] in patients with benign intestinal obstructions and >5.72 (sensitivity, 72%; specificity, 83%) in children with intussusception [15]. In these last patients, resection was associated with a PLR value of >188.5 (sensitivity, 0.80; specificity, 0.69).

Although our value for PLR is similar, the NLR cutoff is sensibly lower than those previously reported. For the WBC, NLR, and SII thresholds, our findings better align with the results obtained by studies aimed at distinguishing between surgical reduction and resection in patients with incarcerated abdominal wall hernias. In these studies, thresholds of 12 for NLR (specificity, 15.2%; sensitivity, 92.9%), 314.5 for PLR (specificity, 37%; sensitivity, 88.3%), 2401.6 for SII (specificity, 78.6%; sensitivity, 45.7%) [11], 18.5 × 10^3^/µL for WBCs (sensitivity, 84.09%; specificity, 50%), and 8.1 × 10^3^/µL for neutrophils (sensitivity, 72.07%; specificity, 65.38%) [9] have been reported. Our results demonstrated even higher specificity for NLR and greater sensitivity for SII and WBCs.

Dogs with perforations were excluded to minimize potential biases from the effects of peritonitis on blood counts. Still, our study has several limitations. Firstly, as a single-center retrospective study, the sample size was small, and selection bias was unavoidable. Larger prospective studies are needed to confirm the prognostic value of these parameters. Secondly, as discussed, blood sampling timing can affect the predictive accuracy of the hematological markers [8]. Thirdly, treatments administered by the referring veterinarian might influence the markers. However, we decided to include dogs treated with antiemetics, antidiarrheal, probiotics, and opioids before diagnosis at the VTH because, to our knowledge, there are no reports of the impact of these drugs on blood cell counts. We also included five dogs receiving antimicrobials, considering that the washout period of a week before admission at the VTH was enough, as demonstrated by the relapse of the clinical signs. These five dogs underwent enterectomy and showed the longest disease duration, but the rest were allocated to both the EO and EE groups. A previous study has shown that dogs receiving prior veterinary treatment are more likely to require EE than those without previous treatment [3]. Still, they considered the presurgery time interval the most important factor for receiving one procedure or the other. They based this conclusion on the fact that both their enterotomy and enterectomy groups included treated and non-treated dogs, and still, the EO group had a shorter duration of illness than the EE group [3], consistent with the findings in the present study. Finally, although these markers may contribute to prognosis, the owner should always be informed about all potential intraoperative and postoperative situations.

## 5. Conclusions

Our findings indicate that the preoperative WBC count, neutrophil count, SII, and particularly NLR are cost-effective, minimally invasive, and readily accessible biomarkers with discriminatory potential regarding the differentiation of enterotomy versus enterectomy in dogs with foreign-body-related intestinal obstruction, which should also be effective for perioperative management. Still, further research is required to validate these findings.

## Figures and Tables

**Table 1:** Hematologic results of the control group of healthy dogs and the dogs with intestinal foreign-body obstruction (FBO) that underwent enterotomy (EO) and enterectomy (EE).

	Control	FBO		
		EO	EE	*p*_EO-EE_
WBC (×10^3^ μL)	8.04 (5.16–11.19; 1.54)	16.95 (7.32–48.41; 9.77)	23.16 (10.31–53.73; 10.15)	0.0001
Lymphocytes (×10^3^ μL)	2.85 (1.65–4.48; 0.69)	1.51 (0.45–2.92; 0.61)	1.07 (0.23–4.00; 0.73)	0.001
Neutrophils (×10^3^ μL)	4.56 (3.26–7.53; 1.22)	13.36 (3.25–41.42; 8.47)	20.32 (9.22–77.00; 11.61)	0.0001
Platelets (×10^3^ μL)	237.5 (148–457; 64.28)	297.5 (78–946; 136.33)	266 (70–729; 125.59)	0.226
NLR	1.85 (0.91–2.93; 0.43)	9.24 (1.50–39.38; 7.29)	18.89 (7.07–75.49; 16.17)	0.0001
PLR	85.93 (44.81–147.24; 31.24)	181.22 (52–704.44; 143.97)	233.80 (17.50–1626.08; 318.10)	0.046
SII (×10^3^)	457.43 (225.62–677.60; 139.21)	2542.26 (385.18–15,989.23; 3392.70)	4832.54 (495.25–27,692.26; 5793.53)	0.0001

WBC: white blood cell, NLR: neutrophils to lymphocytes ratio, PLR: platelets to lymphocytes ratio, SII: systemic immune-inflammation index. Data are shown as median and range (max–min; standard deviation).

**Table 2:** Receiver operating characteristic (ROC) curve analysis, showing the overall accuracies of the blood cell- and hematological-derived markers used to distinguish enterotomy from enterectomy.

	AUC (95% CI)	*p*-Value	Cutoff	Sensitivity (%)	Specificity (%)
WBC (×10^3^ μL)	0.712 (0.609–0.814)	0.0001	19.8	76.2	66.7
Neutrophils (×10^3^ μL)	0.774 (0.683–0.865)	0.0001	14.1	95.3	58.3
Lymphocytes (×10^3^ μL)	0.299 (0.195–0.404)	0.001	1.1	38.1	21.7
Platelets (×10^3^ μL)	0.429 (0.314–0.544)	0.226	489.0	9.5	95.0
NLR	0.827 (0.747–0.908)	0.0001	12.7	90.9	71,7
PLR	0.618 (0.508–0.729)	0.048	172.1	78,5	45.0
SII (×10^3^ μL)	0.756 (0.660–0.851)	0.0001	2812.5	90.4	60

WBC: white blood cell, NLR: neutrophil-to-lymphocyte ratio, PLR: platelet-to-lymphocyte ratio, SII: systemic immune-inflammation index, AUC: area under the curve.

## Data Availability

The data presented in this study are available on request from the corresponding author.
